# Prescribing competency assessment for Canadian medical students: a pilot evaluation

**Published:** 2019-03-13

**Authors:** Anne Holbrook, J. Tiger Liu, Michael Rieder, Michelle Gibson, Mitchell Levine, Gary Foster, Dan Perri, Simon Maxwell

**Affiliations:** 1 Division of Clinical Pharmacology, St. Joseph’s Healthcare Hamilton, Ontario, Canada; 2 Division of Clinical Pharmacology & Toxicology, Department of Medicine, McMaster University, Ontario, Canada; 3 Department of Health Research, Evidence, and Impact, McMaster University, Ontario, Canada; 4 Department of Paediatrics, Physiology & Pharmacology and Medicine, Schulich School of Medicine & Dentistry, Western University, Ontario, Canada; 5 Department of Medicine, Queen’s University, Ontario, Canada; 6 Clinical Pharmacology Unit, University of Edinburgh, Scotland

## Abstract

**Background:**

The knowledge and ability to prescribe safely and effectively is a core competency for every graduating medical student. Our previous research suggested concerns about medical student prescribing abilities, and interest in a standardized assessment process.

**Methods:**

A multi-year cross-sectional study evaluating the feasibility, acceptability, and discriminative ability of an online prescribing competency assessment for final year Canadian medical students was conducted. Students at nine sites of four Ontario medical schools were invited to participate in an online one-hour exam of eight domains related to prescribing safely. Student feedback on perceived fairness, clarity, and ease of use formed the primary outcome. Exam performance and parity between schools were the secondary outcome.

**Results:**

A total of 714 students completed the assessment during spring final review courses between 2016 and 2018. Student feedback was more favourable than not for appropriateness of content (53.5% agreement vs 18.3% disagreement), clarity of questions (65.5% agreement vs 11.6% disagreement), question layout and presentation (70.8% agreement vs 12.2% disagreement), and ease of use of online interface (67.1% agreement vs 13.6% disagreement). Few (23.6% believed their course work had prepared them for the assessment. Mean total exam score was 70.0% overall (SD 10.4%), with 47.6% scoring at or above the pass threshold of 70%.

**Conclusion:** Our prescribing competency assessment proved feasible, acceptable, and discriminative, and indicated a need for better medical school training to improve prescribing competency. Further evaluation in a larger sample of medical schools is warranted.

## Introduction

Every physician, regardless of specialty, must learn clinical pharmacology, therapeutics, and competent prescribing skills. In Canada, doctors write more than 500 million prescriptions annually for more than 1100 prescription drug families.^1^ However, prescribed medications are a common source of adverse events. Medication errors are estimated to be the fifth or sixth leading cause of death.^2^^,^^3^ Prescribing safely involves a combination of complex tasks: 1) gathering an appropriate history from the patient and their medical records; 2) performing a relevant physical exam; 3) applying knowledge to understand whether any medication is indicated, which medication would be most cost-effective for the individual patient’s benefit-harm profile; and 4) creating and communicating a coherent, legible prescription with a practical plan to monitor the patient’s progress.^4^ Both the literature and observations of senior physicians suggest that newly licenced physicians are having difficulties keeping up with knowledge and skills of clinical pharmacology.^5^^,^^6^ This is understandable since this specialty is arguably the most rapidly advancing area of medicine.^7^^-9^ In addition, poor performance on tests of clinical decision-making or communication has been shown to predict patient complaints and disciplinary action over subsequent years of practice.^10^ All of this is of sufficient concern that medical educators in Canada and the United States are creating a required Entrustable Professional Activity based on the ability to write a high quality prescription and counsel accordingly, and Canadian prescribing competencies have been developed in collaboration with the Royal College of Physicians and Surgeons of Canada.^11^

These concerns are not isolated to North American medical graduates. The EQUIP study in England reviewed 124,260 medication orders, finding an error rate as high as 10.3% among junior housestaff.^9^ In response to this study and a series of serious medication safety incidents, education leaders in clinical pharmacology and the British Pharmacological Society instituted the Prescribing Safety Assessment (PSA), an online examination that final year medical students must pass prior to their licensing exams.^12^ Drawing on a large database of validated questions, the full PSA consists of 60 questions and lasts for two hours.^13^ Students participate in the PSA at the end of medical school a few months before their medical licensure examinations, and are encouraged to prepare using practice resources.^14^ Approximately 53,000 students have completed the exam in the last five years, with very positive feedback on fairness and the helpfulness of the exam to their competence to practice.^9^^,^^15^^,16^ These students expressed appreciation for the initiative taken to address the prescription competency concerns, for increased confidence when prescribing as a first year resident, and for making them aware of key prescribing resources such as formularies.^13^^,^^15^^,^^16^

Clinical Pharmacology and Toxicology (CPT) is a much younger, smaller, and less standardized specialty in Canada compared to its UK counterpart of Clinical Pharmacology and Therapeutics. Within the CanMEDS framework of medical education, CPT knowledge and prescribing skills competencies require skills within each and every CanMEDS domain (Scholar, Collaborator, Communicator, Advocate, Leader, Professional) including the more general Medical Expert umbrella.^17^ Indeed, small studies at McMaster University have suggested that medical students perceive CPT training to be important but inadequate, and find an online assessment acceptable.^18^^,^^19^ A subsequent survey of medical education leaders in all 17 of the Canadian medical schools showed that faculty had substantial concerns about the prescribing abilities of a sizable minority of their medical student graduates and junior residents, and expressed strong interest in the incorporation of a standardized prescribing assessment into the medical curricula and licensing process.^20^

Our objectives were to test the technical feasibility, acceptability, and discriminative ability of a Canadian version of the Prescribing Safety Assessment (C-PSA) and use it to gauge the prescribing competence of final year Canadian medical students.

## Methods

We designed a cross-sectional study. In collaboration with the British Pharmacological Society, we developed a one-hour, 30 question PSA and adapted it to common Canadian scenarios and medical terminology. The C-PSA covered eight essential domains of prescribing competence (see Table 1).^21^ Each question had been previously analyzed using a modified Angoff technique for level of difficulty, a method similar to the Bookmark method of validation employed by the Medical Council of Canada for their qualifying examinations.^22^^,^^23^

**Table 1:** Prescribing safety assessment question domains

**Prescription Writing**	Writing a prescription requiring decisions regarding specific drug, dose, route and frequency based on clinical circumstances and supplementary information.
**Prescription Review**	Deciding which components of the current prescription list are inappropriate, unsafe, or ineffective based on clinical circumstances.
**Planning Management**	Deciding which combination of therapies would be the most appropriate to manage a particular clinical situation.
**Providing Information**	Deciding which are the important pieces of information that should be provided to patients to allow them to choose whether to take the medication, or to enhance its safety and effectiveness.
**Calculation Skills**	Making an accurate drug dosage calculation based on numerical information.
**Adverse Drug Reactions**	Identifying likely adverse reactions of specific drugs, drugs that are likely to be causing specific adverse drug reactions, potentially dangerous drug interactions and deciding on the best approach to managing a clinical presentation that results from the adverse effects of a drug.
**Drug Monitoring**	Deciding on how to monitor the beneficial and harmful effects of medicines.
**Data Interpretation**	Deciding on the meaning of the results of investigations as they relate to decisions about on-going drug therapy.

All nine sites at four medical schools agreed to pilot the assessment as part of their Medical Council of Canada Qualifying Examination Part I (MCCQE) review courses for final year medical students in the springs of 2016, 2017, and 2018. All final year medical students at the four schools were contacted by email and invited to participate, with assurances that scores on the C-PSA were not part of their final transcripts. Individual registration was required to assign an anonymous study ID number and to send individuals their own confidential scores. We provided information on the scope of the assessment, question domains, and a mock exam to students on the PSA website *(**https://prescribing**safetyassessment.ac.uk/resources)* prior to the assessment.

Each participating university’s research ethics board reviewed and approved the project.

At the time of the exam for each school, each student signed on to their pre-registered account simultaneously in a classroom setting using wireless connections. Students were provided and encouraged to utilize a direct link to a standardized medical and drug information database, RxTx, as part of the exam interface during the exam (i.e., an “open book” exam).^24^^,^^25^ At the end of the assessment, each participant completed feedback questions on the test’s appropriateness, difficulty level, fairness, time frame, layout, ease of use, and clarity.

Feedback obtained from the students on the assessment, particularly acceptability based on perceived fairness, clarity, and ease of use (details below), was set as the primary outcome of this study. Student total scores, scores on each domain, and whether student performance was comparable between the different medical schools were defined as the secondary outcomes. Based on prior use of the questions with British medical students but allowing for the abbreviated preparation period and shorter exam, we set the exam pass threshold at 70%.

Analyses were largely descriptive. We analyzed performances across test domains using Welch’s t-test and total exam scores across schools using ANOVA. Sample size estimates suggested that 278 participants would be required to have 95% power to detect a 70% plus or minus 5% approval rating on the feedback questions. We used IBM SPSS Statistics 20 for statistical analyses.

## Results

Seven hundred and fourteen students registered for the exam: 259 (approximately 60% of eligible) from School 1, 330 (approximately 97% of eligible) from School 2, 103 (100% of eligible) from School 3, and 22 (approximately 10% of eligible) from School 4. Registration occurred later than expected, only two weeks before the assessments. Twelve (1.7%) students did not enter any responses and we excluded their data from further analyses. We were able to provide marks to all students within 24 hours.

The primary outcome, learner feedback on the exam questionnaire (details in Table A1 in Appendix A), was consistently and substantially more positive than negative - appropriateness of content (53.5% agreement vs 18.3% disagreement), time provided (61.3% agreement vs 21.7% disagreement), question layout and presentation (70.8% agreement vs 12.2% disagreement), ease of use of online interface (67.1% agreement vs 13.6% disagreement), and clarity of questions (65.5% agreement vs 11.6% disagreement). Ratings for helpfulness of the preparation resources at the PSA website, which were primarily British, were moderate with 269 (47.4%) finding them helpful.

Only 134 (23.6%) students agreed that their medical school coursework had prepared them for the assessment. Based on 568 (79.6%) responses, 202 students (35.6%) reported writing ten or fewer prescriptions during their entire training. Students were generally not in favour of making a C-PSA a mandatory requirement to pass prior to licensing exams.

Table A1 (Appendix A) also displays exam scores overall, by domain and by school. Mean total score was 70.0% (SD 10.5%; range 5% to 96%.) with 47.6% of students meeting the suggested overall pass score of 70%. There were significant differences between some of the score domains and between schools (p < 0.05).

## Discussion

This assessment of prescribing competency among final year medical students is the first of its kind in Canada, and the first time that the PSA was hosted wirelessly anywhere. The lack of technical difficulty and the rapid turnaround of marks support the feasibility of this formative assessment of prescribing competency internationally. However, there was a significant difference in overall performance scores between question domains. The lack of preparation time at all schools, no doubt, affected ratings and scores, but this study was meant to be a partial validation and anchoring exercise. The clarity of the assessment, the time provided, and the interface were all acceptable. However, only a few students felt that their medical school training had adequately prepared them for the content in the assessment, and the perceived training gap was reflected in the test scores. The overall pass rate of 47.6% suggests that current training for prescribing competency may be inadequate. Estimates of mandatory teaching time for clinical pharmacology in Ontario medical schools range from 15 to 55 hours in total. Students from the school with the highest number of mandatory teaching hours were the most likely to feel their coursework prepared them for the assessment, and scored the highest overall.

We acknowledge several limitations to our study. First, only four Canadian medical schools were included, with low recruitment success at one of the schools. Second, the pass threshold of 70% was determined indirectly using the PSA experience with British medical students, although we had good reasons to expect that prescribing competency thresholds would be similar. Third, we provided our participants no specific preparation for their C-PSA assessment, and we were unable to provide a good, general prescribing resource online during the exam since there is no Canadian national formulary. Lastly, the exam platform (ASP.Net MVC and PostgreSQL) anonymized the feedback questionnaire responses, therefore we could not measure the correlation between the number of prescriptions and assessment performance. We plan to remedy this particular limitation for future assessments.

### Conclusion

In conclusion, we have demonstrated that a prescribing competency assessment format used regularly in the UK can be adapted and successfully applied in Canada. Our results suggest that graduating medical students are not practice-ready and have not achieved prescribing competency. Further, we recommend testing the assessment across Canada to first determine the scalability of implementation and second to determine medical student prescribing competence more broadly.

## Appendix A

**Table A1:** Feedback and C-PSA score results

		School 1 (n = 229)				School 2 (n = 219)				School 3 (n = 100)				School 4 (n = 20)				Combined (n = 568)			
**The number of prescriptions that I have written on a prescription chart during my training is:**																					
		Number		%		Number		%		Number		%		Number		%		Number		%	
0-5		25		10.92%		82		37.44%		8		8.00%		2		10.00%		117		20.60%	
6-10		29		12.66%		40		18.26%		8		8.00%		8		40.00%		85		14.96%	
11-20		41		17.90%		39		17.81%		27		27.00%		4		20.00%		111		19.54%	
21-50		61		26.64%		34		15.53%		34		34.00%		6		30.00%		135		23.77%	
More than 50		73		31.88%		24		10.96%		23		23.00%		0		0.00%		120		21.13%	
**Student Feedback Questions**																					
*This assessment was an appropriate test of the prescribing skills expected of a medical student upon graduation*	Strongly Disagree	4		1.75%		22		10.05%		7		7.00%		0		0.00%		33		5.81%	
	Disagree	20		8.73%		42		19.18%		9		9.00%		0		0.00%		71		12.50%	
	Neutral	71		31.00%		63		28.77%		24		24.00%		2		10.00%		160		28.17%	
	Agree	122		53.28%		85		38.81%		53		53.00%		18		90.00%		278		48.94%	
	Strongly Agree	12		5.24%		7		3.20%		7		7.00%		0		0.00%		26		4.58%	
*The time provided for answering the questions was sufficient*	Strongly Disagree	2		0.87%		31		14.16%		6		6.00%		0		0.00%		39		6.87%	
	Disagree	7		3.06%		66		30.14%		9		9.00%		2		10.00%		84		14.79%	
	Neutral	31		13.54%		44		20.09%		20		20.00%		2		10.00%		97		17.08%	
	Agree	144		62.88%		68		31.05%		62		62.00%		13		65.00%		287		50.53%	
	Strongly Agree	45		19.65%		10		4.57%		3		3.00%		3		15.00%		61		10.74%	
*The layout and presentation of the questions was easy to follow*	Strongly Disagree	7		3.06%		10		4.57%		4		4.00%		0		0.00%		21		3.70%	
	Disagree	26		11.35%		10		4.57%		12		12.00%		0		0.00%		48		8.45%	
	Neutral	36		15.72%		40		18.26%		19		19.00%		2		10.00%		97		17.08%	
	Agree	127		55.46%		124		56.62%		60		60.00%		13		65.00%		324		57.04%	
	Strongly Agree	33		14.41%		35		15.98%		5		5.00%		5		25.00%		78		13.73%	
*The online interface was easy to use*	Strongly Disagree	6		2.62%		10		4.57%		12		12.00%		0		0.00%		28		4.93%	
	Disagree	17		7.42%		11		5.02%		21		21.00%		0		0.00%		49		8.63%	
	Neutral	47		20.52%		36		16.44%		21		21.00%		6		30.00%		110		19.37%	
	Agree	122		53.28%		126		57.53%		42		42.00%		11		55.00%		301		52.99%	
	Strongly Agree	37		16.16%		36		16.44%		4		4.00%		3		15.00%		80		14.08%	
*The questions in the assessment were clear and unambiguous*	Strongly Disagree	3		1.31%		10		4.57%		4		4.00%		0		0.00%		17		2.99%	
	Disagree	15		6.55%		25		11.42%		8		8.00%		1		5.00%		49		8.63%	
	Neutral	47		20.52%		59		26.94%		22		22.00%		2		10.00%		130		22.89%	
	Agree	137		59.83%		109		49.77%		59		59.00%		14		70.00%		319		56.16%	
	Strongly Agree	27		11.79%		16		7.31%		7		7.00%		3		15.00%		53		9.33%	
*The background and practice information about the PSA was helpful*	Strongly Disagree	4		1.75%		20		9.13%		3		3.00%		0		0.00%		27		4.75%	
	Disagree	15		6.55%		22		10.05%		10		10.00%		0		0.00%		47		8.27%	
	Neutral	125		54.15%		57		26.03%		37		37.00%		7		35.00%		225		39.61%	
	Agree	79		34.50%		97		44.29%		47		47.00%		13		65.00%		236		41.55%	
	Strongly Agree	7		3.06%		23		10.50%		3		3.00%		0		0.00%		33		5.81%	
*My course prepared me for the content of the questions in this assessment*	Strongly Disagree	18		7.86%		49		22.37%		7		7.00%		0		0.00%		74		13.03%	
	Disagree	74		32.31%		79		36.07%		14		14.00%		4		20.00%		171		30.11%	
	Neutral	89		38.86%		59		26.94%		34		34.00%		7		35.00%		189		33.27%	
	Agree	46		20.09%		29		13.24%		42		42.00%		9		45.00%		126		22.18%	
	Strongly Agree	2		0.87%		3		1.37%		3		3.00%		0		0.00%		8		1.41%	
		**School 1 (n = 157)**				**School 2 (n = 155)**				**School 3 (n = 0) ***				**School 4 (n = 20)**				**Combined (n = 332)**			
*An assessment such as this should be mandatory to pass before a Canadian medical student tries the MCCQE-Part1*	Strongly Disagree	30		19.00%		30		19.00%						0		0.00%		60		18.07%	
	Disagree	34		21.00%		34		21.00%						4		20.00%		72		21.69%	
	Neutral	45		29.00%		45		29.00%						9		45.00%		99		29.82%	
	Agree	38		24.00%		38		24.00%						6		30.00%		82		24.70%	
	Strongly Agree	8		5.00%		8		5.00%						1		5.00%		17		5.12%	
**Canadian Prescribing Safety Scores - Question Domain and Total**																					
		**School 1 (n = 247)**				**School 2 (n = 330)**				**School 3 (n = 103)**				**School 4 (n = 22)**				**Combined (n = 702)**			
		Mean	Median		SD	Mean	Median		SD	Mean	Median		SD	Mean	Median		SD	Mean	Median		SD
Prescription Writing		66.87%	67.50%		17.52%	69.46%	70.00%		16.22%	77.01%	80.00%		12.37%	77.61%	75.00%		10.59%	69.91%	70.00%		16.41%
Prescription Review		70.55%	68.75%		13.43%	75.19%	75.00%		11.15%	69.05%	68.75%		12.14%	71.31%	68.75%		10.50%	72.53%	75.00%		12.37%
Planning Management		62.85%	75.00%		23.08%	67.65%	75.00%		18.21%	30.58%	25.00%		20.09%	61.36%	50.00%		20.01%	60.33%	50.00%		23.89%
Providing Information		82.73%	100.00%		22.46%	86.16%	100.00%		19.29%	85.34%	100.00%		20.70%	87.88%	100.00%		19.37%	84.90%	100.00%		20.69%
Adverse Drug Reactions		73.38%	75.00%		22.71%	85.23%	100.00%		17.85%	74.03%	75.00%		24.98%	72.73%	75.00%		15.25%	79.02%	75.00%		21.51%
Drug Monitoring		56.68%	50.00%		24.30%	70.15%	75.00%		26.71%	65.53%	75.00%		21.33%	72.73%	75.00%		21.70%	64.81%	75.00%		25.71%
Data Interpretation		45.07%	33.33%		27.58%	56.46%	66.67%		26.00%	49.51%	33.33%		27.56%	45.45%	50.00%		34.95%	51.09%	66.67%		27.55%
Calculation Skills		72.87%	75.00%		25.16%	76.82%	75.00%		20.08%	72.57%	75.00%		23.61%	64.77%	75.00%		29.54%	74.43%	75.00%		22.93%
Total		66.97%	67.00%		11.09%	72.36%	72.50%		10.02%	69.37%	70.00%		9.05%	72.18%	70.50%		7.53%	70.02%	70.00%		10.48%
